# The role of RNA-binding proteins in orchestrating germline development in *Caenorhabditis elegans*

**DOI:** 10.3389/fcell.2022.1094295

**Published:** 2023-01-04

**Authors:** Mennatallah M. Y. Albarqi, Sean P. Ryder

**Affiliations:** Department of Biochemistry and Molecular Biotechnology, University of Massachusetts Chan Medical School, Worcester, MA, United States

**Keywords:** RNA-binding proteins, nematode, germline, post-transcriptional regulation, embryogenesis, oogenesis, translation

## Abstract

RNA passed from parents to progeny controls several aspects of early development. The germline of the free-living nematode *Caenorhabditis elegans* contains many families of evolutionarily conserved RNA-binding proteins (RBPs) that target the untranslated regions of mRNA transcripts to regulate their translation and stability. In this review, we summarize what is known about the binding specificity of *C. elegans* germline RNA-binding proteins and the mechanisms of mRNA regulation that contribute to their function. We examine the emerging role of miRNAs in translational regulation of germline and embryo development. We also provide an overview of current technology that can be used to address the gaps in our understanding of RBP regulation of mRNAs. Finally, we present a hypothetical model wherein multiple 3′UTR-mediated regulatory processes contribute to pattern formation in the germline to ensure the proper and timely localization of germline proteins and thus a functional reproductive system.

## Introduction

In sexually reproducing organisms, post-transcriptional regulation of maternal and paternal mRNAs is crucial to gametogenesis and early embryo development (Wickens, 1990; Richter, 1991; Curtis et al., 1995; DeRenzo and Seydoux, 2004; Farley and Ryder, 2008; Kang and Han, 2011). During embryogenesis, control of the levels and localization of specific mRNAs and proteins is key for axis formation and cell fate specification (Zhang et al., 2014). Components of these regulatory processes include RNA-binding proteins (RBPs) and/or regulatory small RNAs that affect the stability of the mRNA transcript and ultimately influence protein levels (Shaw et al., 2010). Delineating the mechanisms of post-transcriptional regulation and how they mediate transfer of information from parent to progeny at the mRNA level will provide a deeper understanding of reproduction and early development as well as how misregulation of such processes can lead to disease.

*C. elegans* is a well-suited model organism to study the biology of RBP-mRNA networks and how they regulate early development. They have a large brood size (∼300), develop from an egg to an adult in a short time (2–3 days), and are self-fertile (Corsi et al., 2015). Not only is this nematode easy to grow and maintain, but there is a plethora of genetic and biochemical techniques that enable addressing important questions in the field of RNA regulation in this organism. CRISPR/Cas9 genome editing is straightforward in the worm and has become a standard technique for genetic manipulation (Kim and Colaiacovo, 2019; Vicencio and Ceron, 2021). Many of *C. elegans* developmental and molecular phenotypes are easy to score and analyze (Corsi et al., 2015). Additionally, both *in vitro* and *in vivo* biochemical methods have been developed to study many aspects of protein-RNA interactions (Ryder et al., 2004; Jedamzik and Eckmann, 2009; Jungkamp et al., 2011; Wright et al., 2011; Ryder, 2016).

**FIGURE 1:**
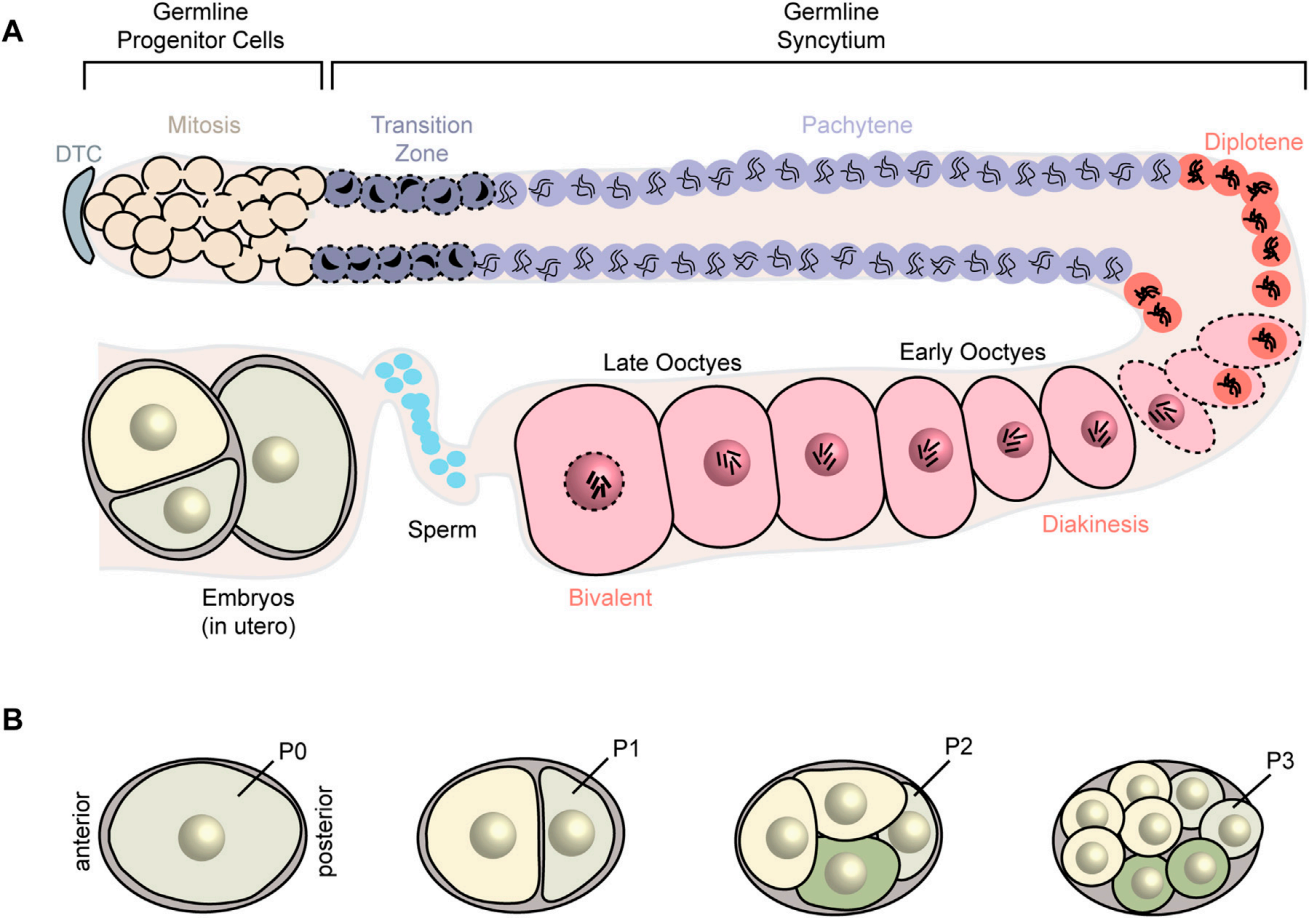
Schematic of *C. elegans* germline and early embryonic stages. **(A)** *C. elegans* gonads consist of two symmetrical arms (one arm represented). In each arm, a distal tip cell (DTC) provides the niche for germ cells to mitotically divide. These germ cells begin to enter meiosis I as they move farther from the DTC. The meiotically dividing cells begin to recellularize around the loop region and form early oocytes which then undergo oocyte maturation and fertilization. **(B)** Axis determination and cell fate specification occurs in the early embryo.

*C. elegans* exist as either sperm-producing males or hermaphrodites that produce both sperm and oocytes from the same germline. Hermaphrodites undergo spermatogenesis during the fourth larval stage and switch to oogenesis during adulthood. The gonads consist of two symmetrical arms. Each arm is a tubular syncytium in which all nuclei share a cytoplasm termed the rachis. In each arm, a distal tip cell provides a niche for progenitor germ cells to divide by mitosis and proliferate and then enter meiosis I. These meiotic nuclei start to fully cellularize in the gonadal arm bend and form oocytes (Figure 1A). As the oocytes approach the proximal end, they get fertilized by the sperm, stored in the spermatheca, and then get delivered to the uterus where the 1-cell embryo undergoes multiple cellular divisions before exiting the animal. Posterior/anterior axis determination and cell fate specification take place during early cellular divisions (Figure 1B). After the eggs hatch, the first larval stage (L1) animals undergo four molts before reaching adulthood (Hubbard and Greenstein, 2005; Goupil et al., 2017; Bauer et al., 2021).

RNA-bindings proteins (RBPs) are essential to cellular function. They contribute to every stage in the journey of an RNA transcript from pre-mRNA splicing, mRNA capping and polyadenylation, to post-transcriptional regulation in the cytoplasm. RBPs vary widely in their RNA-binding domain structure, target binding specificity, and biological function (Burd and Dreyfuss, 1994). Many RNA-binding domains such as hnRNP K homology (KH) domain, RNA recognition motifs (RRM), and zinc-finger domains are highly conserved across metazoans. Some RBPs bind single stranded RNA while others bind dsRNA. Some RBPs such as the ribosomal proteins are more universally expressed and are highly abundant. Others-such as RBPs that regulate germline development-are both spatially and temporally restricted in their expression patterns (Burd and Dreyfuss, 1994; Lee and Schedl, 2006; Hentze et al., 2018).

**FIGURE 2:**
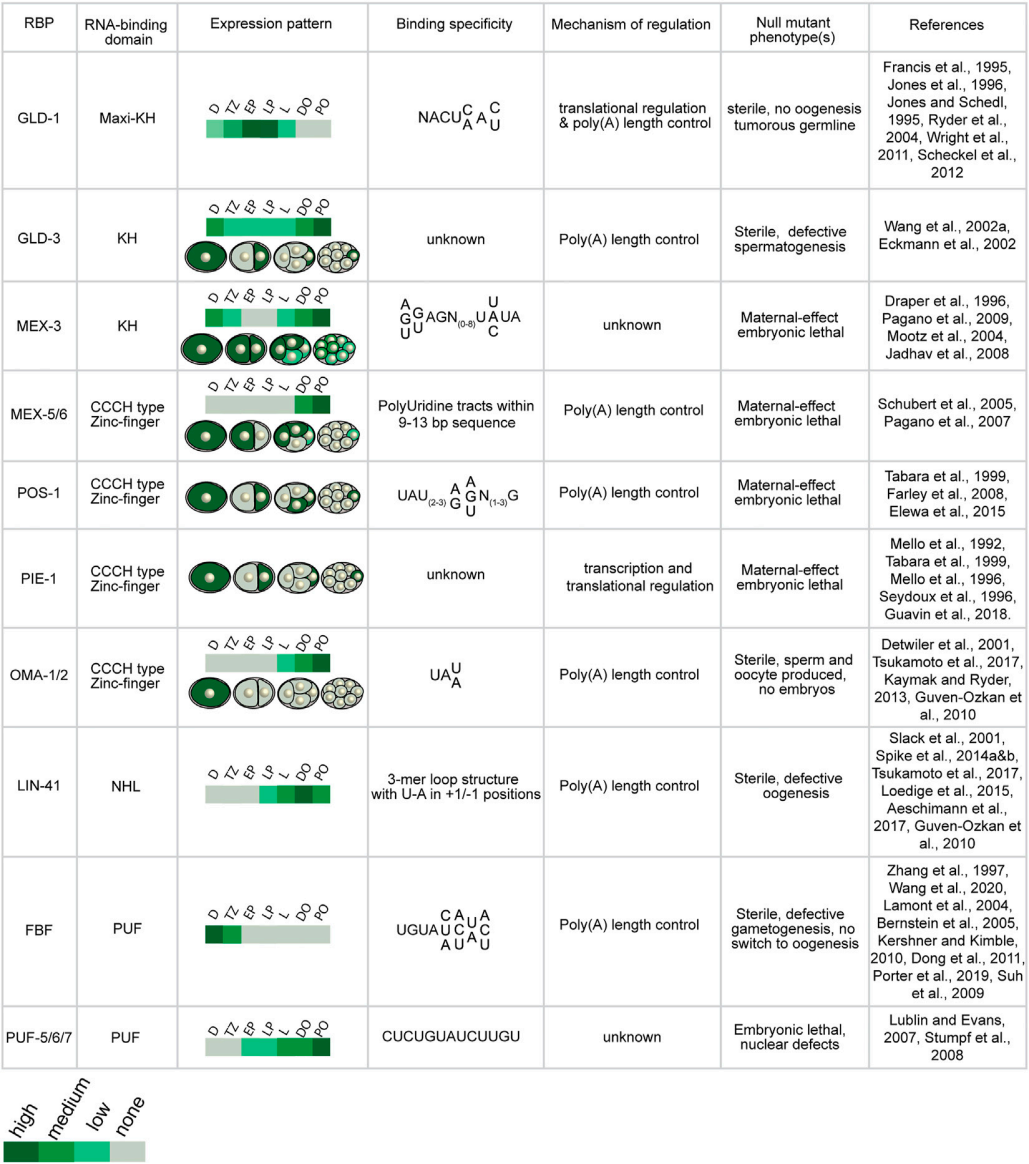
Expression and activities of germline RNA-binding proteins discussed in this manuscript. *C. elegans* germline RBPs vary in their RNA-binding domain, spatiotemporal expression pattern, binding specificity, and mode of post-transcriptional regulation (D: distal, TZ: transition zone, EP: early pachytene, LP: late pachytene, L: loop region, DO: distal oocytes, PO: proximal oocytes).

**FIGURE 3:**
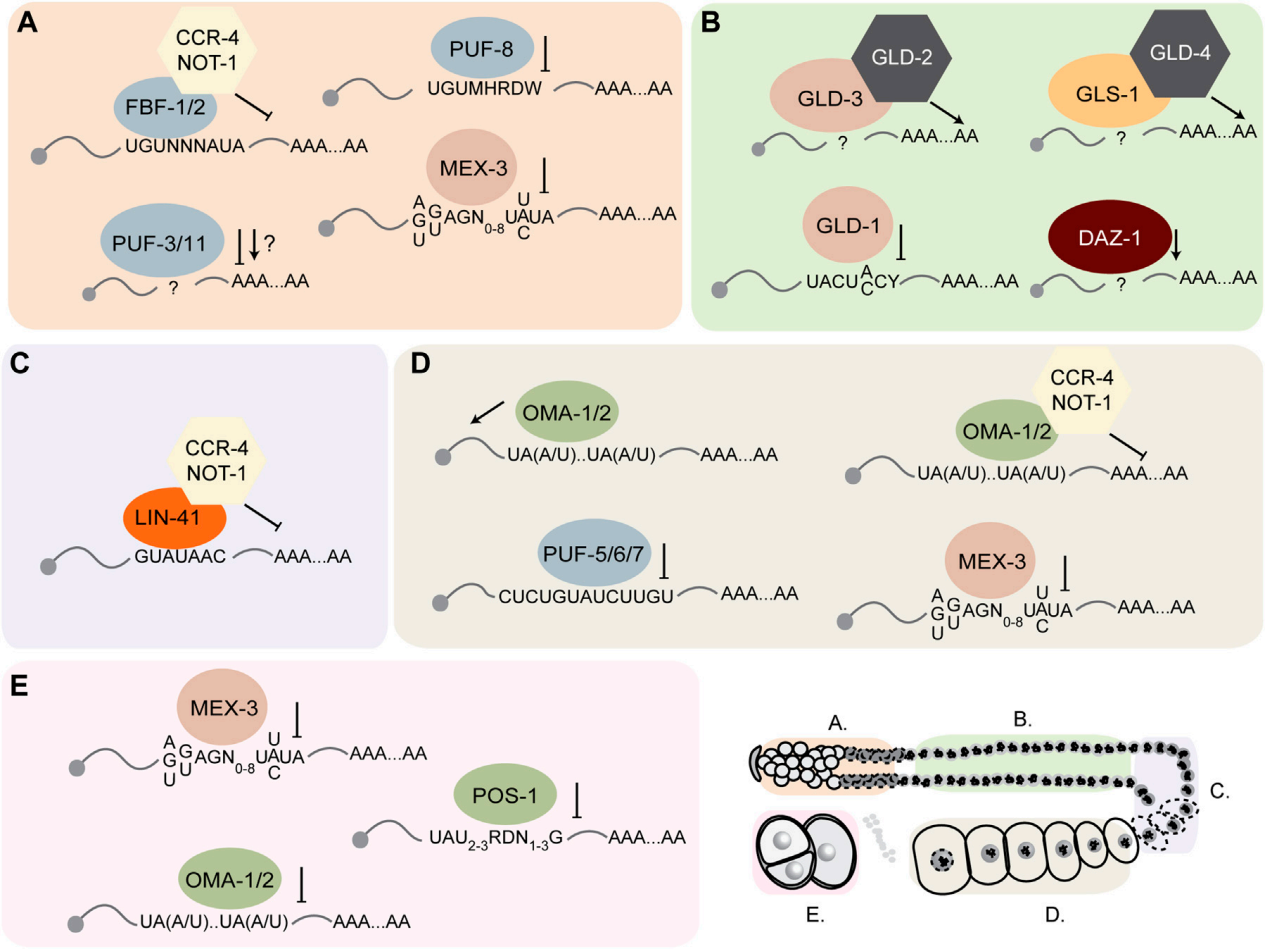
*C. elegans* germline RNA-binding proteins act on common and distinct maternal mRNA targets. In each region of the germline, several RBPs bind specific 3′UTR cis-regulatory elements in their target mRNA to regulate their stability or translational efficiency. **(A-E)** correspond to the region of the germline indicated in the key at the bottom right corner of the figure.

The purpose of this review is to provide an update on how these RBPs direct mRNA regulation during germline development and embryogenesis and outline new approaches that can be used to define their relative importance to reproductive biology. In the past few decades, numerous studies have identified and characterized dozens of germline RNA-binding proteins in *Caenorhabditis elegans* (Figures 2, 3). Many of these proteins contain highly conserved RNA-binding domains that bind the UTRs of their target mRNAs to control different aspects of development including spermatogenesis, spermatogenesis to oogenesis switch in hermaphrodites, oogenesis, and early embryo development (Lee and Schedl, 2006). These germline RBPs can influence the fate of their target mRNAs by modifying their poly(A) tail length and thus mRNA stability, and/or controlling translational efficiency. Together, these RBP-mRNA networks maintain a functional reproductive system required for the animal to effectively reproduce.

### *C. elegans* RNA-binding proteins

The *C. elegans* genome encodes numerous RBPs. One estimate predicts that 887 of *C. elegans* genes encode a protein containing at least one of 17 annotated RNA binding domains (Tamburino et al., 2013). This study, designed to identify all proteins that associate with mRNA in *C. elegans* by interactome capture, detected 594 such RBPs. Many contain evolutionarily conserved domains that are found in other model organisms. For instance, more than half of *C. elegans* RBPs have homologues in *S. cerevisiae* (Matia-Gonzalez et al., 2015). This suggests that post-transcriptional regulation by RBPs is an ancient and evolutionarily conserved mechanism that predates metazoa.

Germline RBPs regulate translation of germline mRNAs through their 3′UTR or 5′UTR to control their spatiotemporal expression pattern (Zhang et al., 1997; Marin and Evans, 2003; Jadhav et al., 2008; Merritt et al., 2008; Suh et al., 2009; Farley and Ryder, 2012; Kaymak and Ryder, 2013; Nousch and Eckmann, 2013; Theil et al., 2018). Intriguingly, transcripts encoding RBPs tend to have long 3′UTRs compared to other genes; the median length estimate is 156 nucleotides compared to the overall average of 129 nucleotides. RBP genes also tend to encode multiple alternatively spliced isoforms (Tamburino et al., 2013). Both features reflect the potential for additional layers of regulation that act at the mRNA level for transcripts that encode regulatory RBPs. RBPs are also frequently regulated at the protein level. They tend to have more phosphorylation sites that can affect their function and activity (Tamburino et al., 2013). For example, the kinase MBK-2 phosphorylates the RBPs MEG-1 and MEG-3 leading to P-granule disassembly (Wang et al., 2014), and the polo-like kinase PLK-1 was shown to phosphorylate the zinc finger RBP POS-1 to promote its diffusion and therefore its asymmetric localization in the early embryo (Han et al., 2018). PAR-4, another kinase, is thought to phosphorylate and inactivate the KH domain-containing RBP MEX-3 during early embryo development to promote soma/germline asymmetry (Huang and Hunter, 2015). RBPs can also be inactivated through ubiquitin-based protein degradation during specific stages of germline development. For instance, the E3 ubiquitin ligase SEL-10 mediates degradation of the RBPs GLD-1, CPB-3 (Kisielnicka et al., 2018), and LIN-41 (Spike et al., 2018). This degradation contributes to localization of the RBP within the germline, which in the case of GLD-1 is critical to its function in promoting the early stages of meiosis. A study by Greenstein and others used CRISPR/Cas9 to target the endogenous locus of the RBP *lin-41* and deleted regions enriched in putative phosphorylation sites. The deletions prevented its degradation in the late oocytes and the early embryo (Spike et al., 2018). Uncovering the role of the phosphorylation sites in the RBP’s localization by mutating them *in vivo* will help define their biological relevance and determine the biological impact on the animal’s development.

### RBPs involved in mitotic progenitor cell self-renewal

In the distal mitotic end of the germline, there are at least six RBPs that promote and maintain mitosis and germ cell proliferation including the PUF-domain RBPs FBF-1/2, PUF-8, PUF-3, PUF-11, and the KH-domain RBP MEX-3 (Figures 2, 3A). There are eleven *C. elegans puf* genes, which were identified based on their sequence homology to the *Drosophila* Pumilio proteins and *C. elegans* FBF proteins (Macdonald, 1992; Zhang et al., 1997; Subramaniam and Seydoux, 2003). The evolutionarily conserved PUF-domain RBPs FBF-1/2 (*fem-3* binding factor) were first discovered in a yeast three-hybrid screen used to identify proteins that bind to the *fem-3* 3′UTR (Zhang et al., 1997). *fbf-1;fbf-2* double mutant animals are sterile and exhibit germline defects including failure to switch to oogenesis and to maintain mitosis. FBF-1/2 contribute to maintenance of mitosis by translationally repressing transcripts encoding the meiosis promoting gene *gld-1* and the Nanos homolog *nos-3* (Hansen et al., 2004). FBF-1/2 also contribute to maintenance of the mitotic region by 3′UTR-mediated repression of genes that encode components of the synaptonemal complex (SC), which forms between homologous chromosomes during meiosis (Merritt and Seydoux, 2010).

Although FBF-1 and FBF-2 are highly similar in their amino acid sequence (∼89%), they are proposed to have opposite functions in the mitotic region and use different mechanisms of post-transcriptional regulation (Lamont et al., 2004; Wang et al., 2020). First, FBF-1 is thought to expand the size of the mitotic region by preventing entry to meiosis while FBF-2 is thought to promote meiotic entry. Null *fbf-1* mutant animals contain less mitotic germ cells than wild type animals while *fbf-2* null mutant animals contain more mitotic germ cells than wild type animals (Lamont et al., 2004). Similarly, *fbf-1* loss of function mutant animals contain a reduced mitotic region while *fbf-2* loss of function mutant animals contain an expanded mitotic region compared to wild type animals (Lamont et al., 2004; Wang et al., 2020). Second, FBF-1 activity requires the CCR4/NOT deadenylase complex while FBF-2 does not (Wang et al., 2020). Knockdown of the CCR-4/NOT complex components in *fbf-1* loss of function mutant animals does not affect the size of the mitotic region but it does lead to a shorter mitotic region in the *fbf-2* loss of function mutant animals. A proposed explanation is that FBF-1 protein contains variable regions that allow it to bind the CCR-4/NOT complex that are absent in FBF-2, despite their high sequence similarity.

It has been shown that the sequence UGU is present within the otherwise variable motifs recognized by PUF proteins. The requirement of this sequence was first discovered by a combination of *in vitro* binding assays with purified recombinant PUF domains and isotopically labeled RNAs (Zamore et al., 1997), and subsequently confirmed in other family members from other species, both *in vitro* and *in vivo* (Nakahata et al., 2001; Tadauchi et al., 2001; Wang X. et al., 2002; Francischini and Quaggio, 2009). The PUF domain in PUF RBPs consists of forty amino acids which constitute eight repeat regions. Each repeat interacts with a base in the consensus binding sequence.

FBF-1/2 binds with high affinity and specificity to the sequence UGUNNNAUA in the 3′UTR of its target mRNAs *gld-1* and *fem-3* as determined by yeast three-hybrid assays and EMSA (Electrophoretic Mobility Shift Assays) (Bernstein et al., 2005) (Figures 2, 3A). Several factors contribute to the ability of FBF-1 and FBF-2 to recognize mRNAs, including their relative abundance, stacking forces between amino acid side chains and nucleotides, hydrogen bonding, steric accommodations, and base flipping. The ideal FBE also contains a cytosine upstream of the core UGU sequence (Bernstein et al., 2005; Kershner and Kimble, 2010; Dong et al., 2011). A study using iCLIP (individual-nucleotide resolution Cross-Linking and ImmunoPrecipitation) identified additional FBF target mRNAs specific to oogenic and spermatogenic germlines (Porter et al., 2019). This study showed that while some FBF targets are present in both male and hermaphroditic germlines, approximately 47% are gender-specific. As expected, some FBF targets encode RNA-binding proteins, supporting a cascade of regulation model where some RBPs govern the post-transcriptional regulation of others during germline development. Identification of all FBF target mRNAs will likely enable prediction of the biological processes in which FBF-1/2 are involved. Many of these targets remain to be validated and their 3′UTRs investigated for functional relevance of the mapped FBEs, and their overall contribution to germline development.

The PUF-domain RBPs PUF-3 and PUF-11 also contribute to maintenance of the mitotic region (Haupt et al., 2020) (Figure 3A). *puf-3;puf-11* double mutant animals are fertile, but loss of both proteins in an *fbf-1;fbf-2* mutant background enhances the mitotic germ cell defects observed in *fbf-1/2* double mutants. PUF-3 and PUF-11 are proposed to act upstream of the meiosis promoting factor GLD-1 (Haupt et al., 2020). RNAi-mediated knockdown of *puf-3* and *puf-11* does not affect the tumorous germline phenotype observed in the *gld-1 gld-2;fbf-1 fbf-2* mutant animals. It is possible that PUF-3 and PUF-11 promote mitosis by repressing meiotic mRNAs in addition to *gld-1*. It is also possible that PUF-3 and PUF-11 act as positive regulators of mitotic mRNAs translation. Revealing the binding specificity of PUF-11 and PUF-3 and mapping their network of *in vivo* targets will help define how these RBPs contribute to maintenance of the mitotic region. PUF-3 and PUF-11 activity could be controlled at the post-transcription or post-translational level by other germline proteins. A recent paper showed that the TRIM-NHL protein NHL-2 promotes meiotic entry by repressing PUF-3 and PUF-11. Expression levels of both proteins appeared increased in the distal mitotic region in *nhl-2* null mutant animals (Brenner et al., 2022).

The final two RBPs that have been shown to contribute to maintenance of mitosis in the distal end are the PUF-domain RBP PUF-8 and the KH-domain RBP MEX-3 (Mootz et al., 2004; Ciosk et al., 2006; Ariz et al., 2009; Mainpal et al., 2011). *mex-3* was first identified in a screen for maternal-effect embryonic lethal mutations (Draper et al., 1996) (Figure 2; Figure 3A). Both MEX-3 and PUF-8 act redundantly in maintenance of germ cell mitosis in the distal end. Single loss of function mutant animals of *puf-8* or *mex-3* contain few germ cells, but *puf-8;mex-3* double mutant animals are sterile. They produce very few germ cells (less than 50) compared to wild type animals (∼430) (Ariz et al., 2009). Additionally, PUF-8 contributes to mitotic germ cell proliferation through 3′UTR-mediated positive regulation of the ER protein FARL-11, which in turn promotes Notch/GLP-1 signaling. MEX-3 is also thought to act redundantly with PUF-8 to promote expression of FARL-11 (Maheshwari et al., 2016).

MEX-3, which contains two KH (K homology) domains, binds two short motifs separated by 0–8 bases (A/G/U) (G/U)AGN_(0–8)_U(U/A/C)UA) as determined by *in vitro* binding studies performed with purified recombinant protein (Pagano et al., 2009). PUF-8 binds UGUMHRDW (M: A/C, H: A/U/C, R: A/G, D: A/U/G, W: A/T) motifs (Opperman et al., 2005; Campbell et al., 2012). It is intriguing to note that there is similarity in the motifs recognized by both proteins, but the *in vivo* relevance of this phenomenon remains unknown. Although it is possible to predict the target mRNAs of MEX-3 and PUF-8 based on the presence of the motifs they recognize, there is limited information concerning which mRNAs are bound by these proteins, and how binding contributes to germ cell proliferation and mitosis (Ariz et al., 2009; Pagano et al., 2009; Mainpal et al., 2011; Datla et al., 2014; Maheshwari et al., 2016). Intriguingly, spatiotemporal expression of MEX-3 is regulated through its 3′UTR in the germline (Merritt et al., 2008; Kaymak et al., 2016). Deleting majority of its 3′UTR at the endogenous locus results in significant de-repression of MEX-3 throughout the germline and a modest reduction in brood size (Albarqi and Ryder, 2021).

Taken together, there is a network of RNA-binding proteins and target mRNAs that promote and maintain germ cell proliferation in the distal end of the germline (Figure 3A). Several PUF proteins, MEX-3, and possibly NHL-2 appear to be required in combination to ensure mitosis in the distal end, presumably through regulation of an overlapping suite of critical mRNA targets. Redundancy is an emerging feature of these RBP-mRNA networks. Having multiple RBPs that act in the same pathway share target mRNAs ensures the repression or activation of gene expression and may improve the robustness of the germline under unfavorable conditions.

### RBPs involved in the mitosis to meiosis switch

In the *C. elegans* germline, multiple RBP-mediated pathways promote the transition from mitosis to meiosis recently reviewed by Pushpa et al. (2017). Two critically important factors are the maxi KH-domain RBP GLD-1 and the RRM-containing RBP DAZ-1 (Figures 2, 3B). *gld-1* was first identified in a mutagenic EMS screen looking for animals with germline defects. Dozens of *gld-1* mutant alleles were identified and characterized using three-factor mapping and Nomarski microscopy. Complete loss of *gld-1* function causes defects in meiotic progression (Francis et al., 1995a). Germ cells exit meiosis, return to mitosis, and continue to proliferate leading to tumor formation and sterility. Other *gld-1* mutants exhibit a wide variety of phenotypes reflecting the various processes controlled by this RBP. For example, some hypomorphic alleles make oocytes that are small and defective (Jones and Schedl, 1995; Jones et al., 1996) indicating a role in oogenesis, while others make only sperm and fail to begin oogenesis. Transgenic reporter studies and immunostaining of dissected germline gonads show that GLD-1 is expressed at very low levels in the distal mitotic end (Jones et al., 1996) (Figure 2). This is presumably due to the negative regulation mediated by FBF-1/2. As FBF-1/2 levels start to decrease in the transition zone, GLD-1 levels increase in the meiotic region where its presence is necessary for promoting meiosis. The expression level decreases again in the loop region and disappears in the oocytes (Jones et al., 1996).

GLD-1 also contributes to maintenance of totipotency of the germline. Dissected gonads of a *gld-1* null mutant express a neuronal fluorescent transgene in the germline. This phenotype is enhanced in a *gld-1;mex-3* double mutant (Ciosk et al., 2006) indicating a role for both GLD-1 and MEX-3 in promoting the stemness of the germ cells. GLD-1 contains a maxi KH domain flanked by two conserved Qua domains. It binds ((U > N)ACU(C/A)AY) motifs in the 3′UTR and/or 5′UTR of its target mRNAs and binds as a homodimer (Lee and Schedl, 2004; Ryder et al., 2004; Wright et al., 2011; Theil et al., 2018) (Figure 2). *In vitro* and *in vivo* binding assays have identified more than 400 targets, majority of which are expressed in the germline and are involved in DNA replication, cell cycle, and mitosis (Lee and Schedl, 2001; Jungkamp et al., 2011). GLD-1 also represses genes that need to be translated only in later stages of germ cell development such as *rme-2* (receptor-mediated endocytosis), a gene that encodes a yolk receptor involved in yolk endocytosis which is part of oocyte growth (Grant and Hirsh, 1999; Lee and Schedl, 2001). The contribution of many GLD-1 associated mRNAs towards the phenotypes observed upon loss of *gld-1* function remains to be determined.

Deleted in azoospermia (*daz-1*) is a homologue of the conserved mammalian DAZL and *Drosophila* Boule RNA-binding proteins. DAZ-1 is required for germ cell development during oogenesis but not spermatogenesis. In *daz-1* null mutant animals, germ cell nuclei arrest in the pachytene stage and fail to progress through meiosis leading to sterility (Karashima et al., 2000; Maruyama et al., 2005). It is mainly expressed in the distal mitotic end, transition zone, and at low levels in the meiotic region (Karashima et al., 2000) (Figure 2). DAZ-1 may contribute to meiotic progression by targeting other RNA-binding proteins in the meiotic region. For instance, DAZ-1 is proposed to positively regulate the expression of *gld-1* through its 3′UTR (Theil et al., 2019). Knockdown of *daz-1* results in repression of a *gld-1* 3′UTR reporter in the meiotic region. Interestingly, *daz-1* null mutant animals do not exhibit defects in the mitotic germ cells which could be because DAZ-1 may be acting redundantly with other RBPs in the mitotic region; that loss of DAZ-1 alone does not have a phenotype. The binding specificity of DAZ-1 is unknown. Mapping the binding specificity of DAZ-1 will enable prediction of the network of target mRNAs. Then, genome editing tools can be used to delineate which targets are required for DAZ-1 regulation of meiosis and the mitosis to meiosis switch.

### RBPs involved in spermatogenesis

Spermatogenesis occurs in each gonadal arm in fourth larval stage males and hermaphrodites. While males continue to make sperm, hermaphrodites switch to oogenesis once they become adults. During spermatogenesis in both sexes, the primary spermatocytes are part of a syncytium sharing a cytoplasmic core termed the rachis. As the primary spermatocytes progress through meiosis, they separate from the syncytium and continue to develop and divide to form the secondary spermatocytes. The secondary spermatocytes undergo the second round of meiotic division and develop into spermatids which later become spermatozoa ready to fertilize an oocyte (L'Hernault, 2006).

In both hermaphrodites and males, GLD-1 promotes meiosis. Some *gld-1* mutant alleles result in feminization of the germline (FOG) where the mutant hermaphrodites fail to make sperm and only make oocytes (Francis et al., 1995b). Others result in a masculinized (MOG) phenotype. GLD-1 and GLD-2, a cytoplasmic poly(A) polymerase, function in redundant pathways to promote meiotic entry and progression during spermatogenesis. Animals lacking both proteins fail to enter meiosis in both sexes. In males, GLD-1 and PUF-8 act redundantly to promote meiotic progression. Animals that lack both GLD-1 and PUF-8 form germline tumors in both hermaphrodites and males (Priti and Subramaniam, 2015). These germline RBPs contribute to the robustness of animal development during unfavorable conditions. *puf-8* single mutant males form germline tumors at elevated temperatures but not at room temperature. The meiotic spermatocytes exit meiosis and continue to proliferate.

Additional RNA-binding factors that play a role in sperm fate include the CPEB homolog *fog-1* and the TOB1 homolog *fog-3*, both of which have been shown to co-immunoprecipitate with a wide variety of oogenic mRNAs (Noble et al., 2016). These proteins appear to work in complex, as immunoprecipates reveal association between both factors and an overlap in their associated mRNAs. As with some *gld-1* alleles, loss of either *fog-1* or *fog-3* causes feminization of the germline (Barton and Kimble, 1990; Ellis and Kimble, 1995). The current model proposes that a FOG-1/FOG-3 complex represses oogenic mRNAs to promote sperm fate, although it remains formally possible that they may promote the expression of a spermatogenic mRNA required for sperm fate.

### RBPs involved in the spermatogenesis to oogenesis switch

RNA-binding proteins required for the switch from spermatogenesis to oogenesis include GLD-1, FBF-1/2, NOS-3, PUF-8, MEX-3, DAZ-1, and MOG proteins (Kraemer et al., 1999; Eckmann et al., 2002; Bachorik and Kimble, 2005; Otori et al., 2006; Ariz et al., 2009; Priti and Subramaniam, 2015; Hu et al., 2019) (Figure 2). The phosphatase FEM-3 and the transmembrane protein TRA-2 control this switch in *C. elegans* germline (Ahringer et al., 1992; Goodwin et al., 1993). FEM-3 promotes spermatogenesis while TRA-2 promotes oogenesis. During the fourth larval stage when the animals produce sperm, *tra-2* mRNA is translationally repressed through its 3′UTR by GLD-1 and FOG-2 (Clifford et al., 2000). On the other hand, *fem-3* mRNA is translationally repressed through its 3′UTR by FBF-1/2 and NOS-3 to promote the switch to oogenesis (Kraemer et al., 1999). NOS-3 is a homologue of *Drosophila* Nanos and has been shown to physically interact with FBF-1 in a yeast two-hybrid screen. Mutations in the binding site of FBF-1/2 in the 3′UTR of *fem-3* or knockdown of *fbf-1/2* cause a masculinization phenotype where hermaphrodites make only sperm and fail to switch to oogenesis. *fem-3* mRNA is also regulated by a set of MOG genes (*mog-1*, *mog-2*, *mog-3*, *mog-4*, *mog-5*, *mog-6*). MOG-1, MOG-4, and MOG-5 are DEAH-box containing proteins. Loss of function mutants of any of these six genes causes germline masculinization. (Graham et al., 1993; Graham and Kimble, 1993; Gallegos et al., 1998; Puoti and Kimble, 2000). These proteins are thought to repress *fem-3* mRNA through its 3′UTR as demonstrated by the de-repression of a *fem-3* 3′UTR transgene reporter in the *mog* mutants. While MOG-1, MOG-4, and MOG-5 are orthologues of human and yeast pre-mRNA splicing factors, their role in splicing in *C. elegans* remains poorly understood. *prp-17*, which is an orthologue of human and yeast pre-mRNA splicing factor PRP17/CDC40, was also shown to contribute to the spermatogenesis to oogenesis switch (Kerins et al., 2010). Loss of function mutants of *prp-17* exhibit the germline masculinization phenotype. Additional genetic epistasis studies with other factors in the sex-determination pathway suggest that PRP-17 functions upstream of *fem-3*.

PUF-8 is also proposed to work with FBF-1 to control the switch to oogenesis. *puf-8;fbf-1* double mutant animals have a masculinized germline (Bachorik and Kimble, 2005). However, it is unknown whether they act on the same target mRNAs. MEX-3 also appears to contribute to the spermatogenesis to oogenesis switch. A percentage (34%) of homozygous *puf-8* mutant animals that are also heterozygous for *mex-3* produce sperm but no oocytes. Thus, it is possible that MEX-3 partially contributes to the switch when *puf-8* is compromised (Ariz et al., 2009). DAZ-1 is also proposed to act in the sex determination pathway (Otori et al., 2006). *In vitro* binding assays have shown that DAZ-1 can bind the 3′UTR of *fbf-1/2*. Moreover, FBF-1/2 levels are reduced in null mutants of *daz-1*. And knockdown of *daz-1* in a *fem-3* gain of function mutant background, which make only oocytes, results in sperm production in these mutant animals. DAZ-1 may be acting redundantly with other RBPs to control sex-determination. These observations add another layer of complexity to how RNA-binding proteins vary in their contribution to different developmental processes and mechanisms throughout germline development.

### RBPs involved in oocyte development and maturation

In adult hermaphrodites, nuclei in the loop region of the germline arrest in diakinesis of meiosis I. These nuclei start to re-cellularize in the loop region to form the early oocytes (Hubbard and Greenstein, 2005) (Figure 1A). These oocytes undergo multiple changes that constitute a maturation process that entails breakdown of the nuclear envelope, assembly of meiotic spindles, and chromosome segregation. Several RBPs control meiotic nuclei cellularization in the loop region and oocyte maturation in the proximal germline including LIN-41, OMA-1/2, PUF-5, PUF-6/7, and MEX-3. LIN-41, a TRIM-NHL RNA-binding protein, starts to appear only in the loop region as meiotic nuclei start to re-cellularize (Figure 2). *lin-41* was first identified as part of the heterochronic pathway that regulates temporal larval development (Slack et al., 2000). LIN-41 was shown to be necessary for repression of target mRNAs such as *cdk-1* to prevent pre-mature oocyte maturation (Spike et al., 2014a). Loss of *lin-41* causes de-repression of *cdk-1* resulting in pre-mature cellularization of pachytene nuclei and formation of small defective oocytes (Tsukamoto et al., 2017). Additionally, LIN-41 regulates other germline mRNAs including *zif-1* and *rnp-1* through their 3′UTR (Spike et al., 2014b) (Figure 3C). *lin-29,* which encodes a transcription factor involved in seam cell self-renewal and differentiation, is also translationally repressed by LIN-41 (Aeschimann et al., 2017; Tsukamoto et al., 2017). LIN-41 recognizes a 3-mer loop structure with U-A in the +1/−1 position (Figure 2; Figure 3C). The binding specificity was revealed by RNAcompete, an *in vitro*-based assay (Ray et al., 2009) that was used to identify the binding specificity of several NHL-domain RNA-binding proteins (Loedige et al., 2015).

OMA-1/2 are zinc-finger RNA-binding proteins that are expressed as oocytes form (Detwiler et al., 2001; Tsukamoto et al., 2017). *oma-1* and *oma-2* were identified by analyzing mutants with embryonic defects. Single *oma-1* or *oma-2* mutants are fertile, but *oma-1;oma-2* double mutant animals are sterile indicating redundant function (Detwiler et al., 2001). Oocytes produced by the double mutant animals are abnormally large, exhibit defects in the nuclear envelope, and arrest in diakinesis (Detwiler et al., 2001). Many of these defects are presumably caused by de-repression of OMA-1/2 target mRNAs, including *cdc-25.3*, which encodes a cell cycle phosphatase and is also targeted by LIN-41 (Spike et al., 2014a; Spike et al., 2014b; Tsukamoto et al., 2017). OMA-1/2 contain two CCCH zinc-finger domains that recognize and bind UA (A/U) motifs (Figure 2; Figure 3D) (Kaymak and Ryder, 2013). Among the targets of OMA-1/2 is the *glp-1* 3′UTR which contains several UA (A/U) motifs. Purified recombinant OMA-1 binds fragments of the *glp-1* 3′UTR *in vitro* and knockdown of *oma-1/2* results in increased expression of a *glp-1* 3′UTR transgenic reporter in the oocytes (Kaymak and Ryder, 2013). Interestingly, OMA-1 also binds its target 3′UTRs with high cooperativity, suggesting that the density and distribution of the motifs might contribute to target selection. Analysis of the 3′UTRs of the mRNAs associated with epitope-tagged OMA-1 show enrichment of UA (A/U) motifs (Tsukamoto et al., 2017). OMA-1/2 have been shown to repress numerous 3′UTR reporter mRNAs in maturing oocytes (Kaymak et al., 2016), all of which contain multiple OMA-1 motifs. Both LIN-41 and OMA-1/2 regulate the development of early and late-stage oocytes through 3′UTR mediated translational regulation of target mRNAs (Figures 3C,D).

Epitope tagged LIN-41 and OMA-1 proteins were pulled down and their associated proteins and RNAs analyzed (Tsukamoto et al., 2017). LIN-41 appeared to associate with several germline RNA-binding proteins including OMA-1, GLD-1, MEX-3, and SPN-4. LIN-41 also appeared to associate with components of the cytoplasmic adenylation (GLD-2, GLD-3, RNP-8) and de-adenylation (CCF-1, NTL-1, CCR-4) complexes. Interestingly, GLD-1, MEX-3, SPN-4, and LIN-41 were also shown to associate with OMA-1. While both LIN-41 and OMA-1 appear to associate with similar protein cofactors, null mutants of *lin-41* or *oma-1*/2 exhibit different phenotypes. *lin-41* null mutant animals undergo premature nuclei cellularization (Spike et al., 2014a) while *oma-1/2* null mutant animals undergo normal cellularization but fail to undergo oocyte maturation (Detwiler et al., 2001). LIN-41 and OMA-1/2 also associate with similar mRNA targets including *cdc-25.3* and *zif-1*, both of which are translationally repressed through their 3′UTR by OMA-1/2 and LIN-41 (Guven-Ozkan et al., 2010; Spike et al., 2014b). The current model proposes that LIN-41 promotes oocyte growth and prevents oocyte maturation in the early oocytes in part by 3′UTR-mediated translational repression of mRNAs, some of which are also repressed by OMA-1/2 such as *cdc-25.3*, *zif-1*, and *rnp-1*. One the other hand, *spn-4* and *meg-1* are translationally repressed by LIN-41 in the early oocytes but positively regulated by OMA-1/2 in the late oocytes. Both SPN-4 and MEG-1 are required for early embryo development (Gomes et al., 2001; Huang et al., 2002; Leacock and Reinke, 2008; Kapelle and Reinke, 2011; Huang and Hunter, 2015).

The PUF proteins PUF-5/6/7 also contribute to late oocyte development (Lublin and Evans, 2007) (Figure 3D). *puf-5* and *puf-6/7* were identified in an RNAi screen of genes expressed in the germline to look for additional regulators of *glp-1* mRNA. Immunostaining showed that PUF-5 starts to appear in the loop region and its levels increase throughout oocyte development until oocyte maturation where PUF-5 is absent in the most proximal oocytes. A yeast three-hybrid screen was used to identify the binding specificity of PUF-5 (Stumpf et al., 2008). PUF-5 and PUF-6 bind motifs containing two UGU trinucleotides (CUCUGUAUCUUGU) (Figure 2). Based on this consensus sequence, a set of potential mRNA targets of PUF-5 and PUF-6 were identified using bioinformatic analysis. Whether this sequence is also the consensus sequence *in vivo* remains unknown. PUF-5 and PUF-6/7 are proposed to repress translation of *glp-1* notch receptor mRNA which is expressed in the distal mitotic end and repressed in the pachytene stage of meiosis by GLD-1. *puf-5;puf-6/7* mutant animals contain maturing oocytes of variable size, pachytene nuclei rather than diakinesis nuclei, and exhibit de-repression of *glp-1*. Thus, PUF-5 and PUF6/7 appear to contribute to early stages of oogenesis, not mature oocytes, in part by repressing the mitotic gene *glp-1*. MEX-3 is also present in the maturing oocytes (Figure 2). MEX-3 alone is not essential for oogenesis. Loss of function *mex-3* mutants produce oocytes that can get fertilized and produce embryos but fail to hatch. Interestingly, MEX-3 protein and RNA appears to associate with LIN-41 and OMA-1 (Tsukamoto et al., 2017), but the role of MEX-3 in oocyte maturation, if any, is unknown.

### RBPs involved in cell-fate specification in early embryos

Prior to the onset of zygotic transcription, the early embryo depends on the cytoplasmic content inherited from the gametes and especially the oocyte (Figure 1B). There are several RBPs that ensure proper embryonic development including POS-1, MEX-3, MEX-5/6, PUF-5, and PUF-6/7 (Figures 2, 3). *pos-1* was first identified in a molecular screen looking for mRNAs that exhibit an asymmetric pattern in the embryo and another genetic screen for maternal-effect lethal mutations. Null mutants of *pos-1* are maternal-effect embryonic lethal. These animals produce dead embryos that express GLP-1 in all blastomeres and show cell fate specification defects such as germ cell precursor cells developing into somatic cells (Farley and Ryder, 2012; Elewa et al., 2015). POS-1 is present throughout the 1-cell embryo but then gets restricted to the posterior (P1) blastomere at the 2-cell stage, the EMS and P2 blastomeres at the 4-cell stage, and the P4 germline precursor cell (Figure 2). POS-1 contains two CCCH zinc finger RNA-binding motifs (Tabara et al., 1999). The POS-1 recognition motif (PRE) is UA (U_2-3_)(A/G)(A/G/U)(N_1-3_)G which was determined using *in vitro* binding assays with recombinant POS-1 tandem zinc finger (TZF) domain (Farley et al., 2008). Several studies have shown that POS-1 represses translation of *glp-1* and *skn-1* mRNAs by binding to its target motifs in their 3′UTRs, restricting their expression to the anterior blastomeres where they are required for somatic tissue specification. POS-1 also contributes to establishing the posterior-anterior asymmetry by 3′UTR-mediated repression of *neg-1* mRNA (Elewa et al., 2015). Additionally, POS-1 is required for 3′UTR-dependent *nos-2* expression in the P4 germline precursor cell (Jadhav et al., 2008). Epistasis experiments suggest that POS-1 acts to de-repress negative regulation by MEX-3 and SPN-4 rather than directly activating the translation of *nos-2*. *In vitro* binding assays show that POS-1 competes with SPN-4-mediated repression of *nos-2*. As such, the ratio of POS-1 and SPN-4 influences the ability of POS-1 to de-repress *nos-2* translation in the P4 cell.

PIE-1 is another RNA-binding protein that exhibits an expression pattern similar to that of POS-1. While it appears throughout the entire 1-cell embryo, it only appears in germline precursor cells and their descendants in later embryonic stages (Figure 2). *pie-1* was identified in a screen for maternal-effect mutations that cause defects in early embryo cell fate specification. It produces extra pharyngeal cells in the embryo when mutated (Mello et al., 1992). PIE-1 also contributes to germline specification in the germline precursor cells (Mello et al., 1996; Tabara et al., 1999; Tenenhaus et al., 2001; Elewa et al., 2015; Gauvin et al., 2018) in part by repressing the translation of *skn-1* through its 3′UTR in the P2 blastomere at the 4-cell stage. SKN-1 is required for specification of the somatic endo-mesoderm (EMS) blastomere (Bowerman et al., 1993). In addition to its function in repressing *skn-1*, PIE-1 promotes germline fate by repression of transcription of zygotic mRNAs in the germline blastomeres (Seydoux et al., 1996).

MEX-5/6 are RBPs that first appear in the distal oocytes and continue to be expressed in the 1-cell embryo, the anterior blastomere in the 2 and 4-cell stages and then remain in the P-granules in the posterior blastomere and its descendants (Schubert et al., 2000) (Figure 2). *mex-5* was identified in a screen for maternal-effect lethal mutations. Null mutants of *mex-5* produce eggs that fail to hatch; embryos contain extra muscle cells and undergo morphogenesis defects. EMSA experiments with purified recombinant MEX-5 tandem zinc finger (TZF) showed that MEX-5 consensus binding site is a 9–13 nucleotide sequences with six or more uridines (Pagano et al., 2007). Additionally, recombinant MEX-5 TZF binds the 3′UTR of *glp-1 in vitro*. MEX-5 is thought to positively regulate expression of *glp-1* since it is absent in *mex-5; mex-6* mutant embryos or repress other RBPs that repress *glp-1* (Schubert et al., 2000; Pagano et al., 2007). MEX-5 and MEX-6 have a high degree of similarity in their amino acid sequence (>50%). However, unlike *mex-5*, *mex-6* null mutant animals produce viable eggs that hatch and develop normally. Reduction of *mex-6* in a *mex-5* null mutant background causes embryonic defects that are more severe than those observed in the *mex-5* null mutant alone, indicating that MEX-6 contributes to embryonic development in a manner that can only be visualized in the absence of *mex-5* (Schubert et al., 2000).

MEX-3, which plays a role in mitotic germ cell totipotency, also contributes to anterior cell fate specification in the early embryo (Draper et al., 1996). One key function of MEX-3 is to repress *pal-1* expression through its 3′UTR (Mootz et al., 2004) (Figure 2). PAL-1, a homeodomain transcription factor, is thereby restricted to the posterior blastomeres where it contributes to the somatic tissue specification of muscle cells (Baugh et al., 2005) (Figure 3E). MEX-3 also contributes to early embryonic development by 3′UTR-mediated repression of *nos-2* (Jadhav et al., 2008). PUF-5 and PUF-6/7 also play a role in early embryo development where they are required for cytokinesis, nuclear divisions, and eggshell formation (Lublin and Evans, 2007). Early embryos of *puf-5;puf-6/7* mutant animal lack a fully formed eggshell, contain abnormal nuclei, and exhibit cellularization defects.

Interestingly, the consensus binding motifs of embryonic RBPs tend to overlap, which may be important to their biological activity. For example, the 3′UTR of *glp-1* contains ∼100 bp region that contains overlapping binding sites for POS-1, GLD-1, MEX-3, FBF-1/2, and OMA-1/2 (Marin and Evans, 2003; Farley et al., 2008; Pagano et al., 2009; Kaymak and Ryder, 2013). Mutating GLD-1 binding motif (GBM) or the POS-1 recognition element (PRE) causes de-repression of a *glp-1* 3′UTR transgenic reporter in the posterior blastomere of a four cell embryo (Farley and Ryder, 2012). Although this cluster contains two nearly identical POS-1 sites (PREs) separated by only five nucleotides, only the 3’ site is required for reporter repression in animals, demonstrating that not every RBP binding site is biologically relevant. Similar to how the POS-1 and SPN-4 ratio affects *nos-2* translation, a high POS-1/SPN-4 ratio represses *glp-1* translation (Jadhav et al., 2008). POS-1 which is localized to the posterior blastomeres represses *glp-1*, thereby restricting its expression to the anterior blastomeres (Ogura et al., 2003).

### Some RBPs regulate stability of their target mRNAs through poly(A) tail length control

**FIGURE 4:**
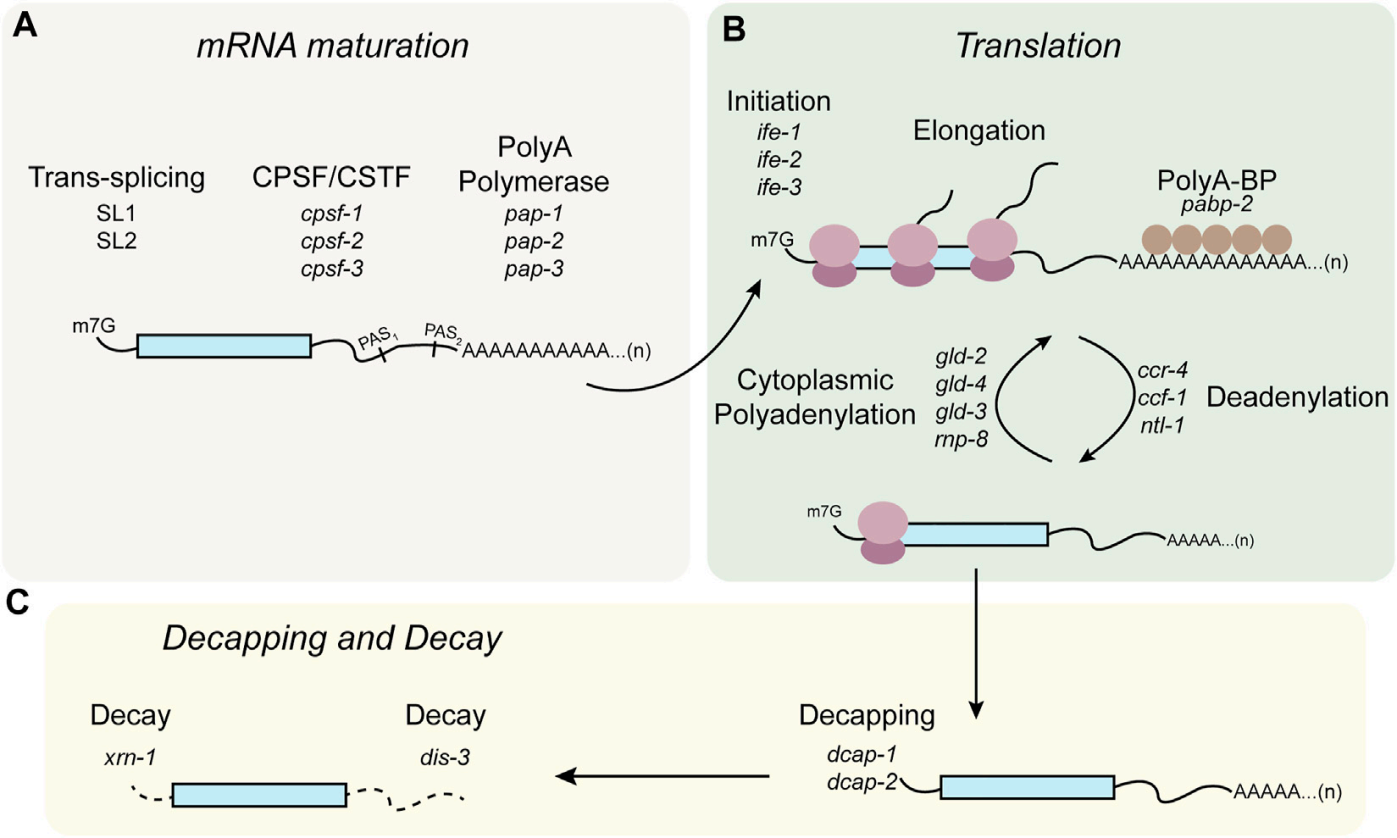
Modes of post-transcriptional regulation in *C. elegans* germline. **(A)** Pre-mRNAs undergo trans-splicing, and alternative polyadenylation. **(B)** In the cytoplasm, mRNAs undergo post-transcriptional regulation mediated through translation initiation or elongation factors, or cycles of polyadenylation and deadenylation. **(C)** mRNAs destined for degradation can undergo 5′ end decapping and decay mediated by exonucleases.

Control of mRNA poly(A) tail length is one of the primary mechanisms of post-transcriptional control in *C. elegans* germline (Figure 4). In the germline, deadenylation of mRNAs does not necessarily lead to mRNA turnover. Two cytoplasmic polyA polymerases (PAP), *gld-2* and *gld-4*, are thought to re-adenylate germline mRNAs to enhance their translation. *gld-2* was identified in a forward mutagenic screen for genes required for germline development, while *gld-4* was identified in a yeast two-hybrid screen (Wang L. et al., 2002; Schmid et al., 2009). Both are part of the DNA polymerase *β*-like superfamily of nucleotidyltransferases, and both are considered non-canonical poly(A) polymerases because they lack an RNA-targeting domain (Wang L. et al., 2002). As such, GLD-2 must be recruited to specific transcripts through interactions with RBPs, for example GLD-3, a multiple KH domain containing RBP shown to interact with GLD-2 in a yeast two-hybrid screen. The complex of GLD-2 and GLD-3 was shown to have robust poly(A) polymerase activity *in vitro* and both are required for germline development (Figure 4B). GLD-2 appears to be targeted to different mRNAs by different interacting partner RBPs. For example, GLD-2 was shown to interact with the RRM-domain containing protein RNP-8. Several genetic and biochemical studies have shown that GLD-2 mediates translational activation mediated by LIN-41 and OMA-1/2 in early stage and late-stage oocytes, respectively (Tsukamoto et al., 2017). The GLD-2/RNP-8 complex also functions to polyadenylate cytoplasmic mRNAs especially in the early stages of oogenesis (Kim et al., 2010). In the 2-cell and 4-cell embryos, POS-1 is thought to repress *neg-1* expression in the posterior blastomere by repressing *gld-2* and *gld-3*, although this specific model has not been tested (Elewa et al., 2015).

Like GLD-2, GLD-4 also lacks an RRM motif to bind its RNA target. GLD-4 binds directly to GLS-1, a P-granule component required for germline survival (Schmid et al., 2009; Nakel et al., 2016) (Figure 4B). In the mitotic region, GLD-4 and its binding partner GLS-1 activate translation of the notch receptor *glp-1* mRNA through poly(A) tail lengthening (Millonigg et al., 2014). Expression of a *glp-1* 3′UTR reporter was shown to be absent in a *gld-4* mutant background. Additionally, the size of the mitotic region was shortened in a *gld-4* or *gls-1* loss of function mutant. Knockdown of *gld-4* also resulted in a shortened poly(A) tail length of the endogenous *glp-1*. GLD-2 and GLD-4 appear to both play redundant and distinct roles in the germline. Null mutant animals of *gld-2* are sterile while *gld-4* null mutant animals exhibit partial sterility meaning that majority of the animals have a reduced brood size, but a small percentage of the animals are completely sterile. Sterility is due to defects in meiotic progression or early stages of oogenesis (Schmid et al., 2009). In the transition zone and as nuclei enter early stages of meiosis, the GLD-2/GLD-3 complex as well as the GLD-4/GLS-1 complex mediate stabilization and translation of *gld-1* mRNA by lengthening its poly(A) tail and repressing de-adenylation (Suh et al., 2006; Schmid et al., 2009).

Shortening of the poly(A) tail *via* de-adenylation also contributes to the post-transcriptional regulation of maternal mRNAs in the germline. The major deadenylase complex consists of CCF-1, CCR-4, and NTL-1 (Nousch et al., 2013) (Figure 4B). These components were identified by homology alignments to the yeast and human genomes in addition to RNAi and immunostaining experiments. CCF-1 is an orthologue of yeast Caf1p (ccr-4 associated factor 1) (Molin and Puisieux, 2005). CCR-4 is an orthologue of yeast Ccr4 (carbon catabolite repressor 4). CCF-1 and CCR-4 carry out the catalytic deadenylase activity while NTL-1 is a scaffolding protein required for assembly of the deadenylase complex. Although all three components are expressed throughout the germline, CCF-1 appears to be most highly expressed in the meiotic region (Nousch et al., 2013). Interestingly, loss of function mutants of *ccf-1* or *ntl-1* but not *ccr-4* lead to reduced fertility that is caused in part by oogenesis defects. The affected animals make small defective oocytes that fail to undergo meiotic maturation. Knockdown of *ccf-1* also delays the expression of OMA-1/2, which are redundantly required for oocyte maturation. In early stages of oogenesis, LIN-41 is thought to repress the translation of its target mRNAs such as *spn-4* and *meg-1* by promoting de-adenylation. All components of the CCR4-NOT complex appear in pull downs with epitope tagged-LIN-41 (Tsukamoto et al., 2017). In the distal mitotic end, FBF-1/2 also appear to repress translation of their target mRNAs, including *gld-1 via* CCF-1-mediated de-adenylation (Suh et al., 2009).

Beyond CCR4-NOT, at least two additional deadenylation complexes exist in the germline, including the PAN deadenylase complex which consists of PANL-2 and PANL-3, and the PARN deadenylase complex comprised of PARN-1 and PARN-2. Loss of function mutants of either *panl-2* or *panl-3* causes a modest reduction in brood size at 20°C but a significantly reduced brood size at 25°C. Loss of function mutants of *parn-1* but not *parn-2* exhibit a reduced brood size at 25°C (Nousch et al., 2013). How they are recruited to specific targets is unknown. The PAN complex as well as the PARN complex are only necessary for germline development and robustness in unfavorable conditions such as elevated temperature and as such are not absolutely required for reproduction. Nevertheless, it is clear that the cycle of cytoplasmic adenylation, governed both by polyadenylation and deadenylation machinery, is critical to RNA regulation in the germline and reproductive fitness (Figure 4B).

### RBPs regulate translation initiation in the germline

Regulation of maternal mRNAs *via* the 3′UTR can also involve translation initiation factor complexes that recognize the 5′ ends of mRNAs (Figure 4B). In *C. elegans*, more than half of the mRNA transcripts have a tri-methylated guanosine (TMG) cap at the 5′ end instead of a mono-methylated guanosine (MMG) cap. This results from a phenomenon in nematodes where one of two splice leader transcripts (SL1/2)) are spliced in trans to pre-messenger RNAs to form the mature 5′ end (Blumenthal, 1998) (Figure 4A). During translation initiation, the cap structure is recognized by eIF4E as part of the assembly process of the pre-initiation complex. Sequence homology-based searches were used to predict homologues of the human eIF4E in the *C. elegans* genome (Keiper et al., 2000). Initially, three isoforms were identified (IFE-1/2/3). Recombinant proteins of these isoforms were purified and used in affinity chromatography assays to determine their ability to bind analogs of the TMG cap. Two additional isoforms were identified later. Knockdown of *ife-1, ife-2, ife-4,* or *ife-5* does not appear to affect viability, but knockdown of *ife-3* causes embryonic lethality (Jankowska-Anyszka et al., 1998; Keiper et al., 2000). This appears to be due to partial redundancy, as knockdown of some isoforms simultaneously also leads to embryonic lethality. For instance, knockdown of both *ife-1* and *ife-2* results in 75% embryonic lethality leading to reduced brood size. The nature of the embryonic lethality remains unknown. *ife-1* null mutant animals exhibit defects in oogenesis and spermatogenesis at higher temperatures and lead to sterility. In these animals, ribosome association of several germline RNA-binding proteins such as *oma-1* appears to be reduced in polysome fractionation (Henderson et al., 2009). Both *ife-1* and *ife-3* are expressed in the germline and contribute to proper gametogenesis. They are thought to act in an opposing fashion to either repress (*ife-3*) or promote (*ife-1*) translational initiation of specific germline mRNAs. IFE-3 is proposed to associate with the RNA-binding proteins FBF and their target mRNAs (Huggins et al., 2020). Knockdown of *ife-3* enhances the masculinization phenotypes of *fbf-1* null mutant animals which supports its proposed role in the sex-determination pathway (Mangio et al., 2015).

Other RBPs are thought to impact translation initiation efficiency, but the mechanisms remain unclear. Polysome profiling of GLD-1 coupled with microarray analysis of GLD-1 targets showed that inhibition of translation initiation is one of the major repression mechanisms used by GLD-1 (Scheckel et al., 2012). Interestingly, GLD-1 may also play a positive role for some of its targets by stabilizing the mRNAs. For example, mutating GLD-1 binding motifs (GBMs) in an *oma-2* 3′UTR reporter causes a reduction in the mRNA levels of the reporter (Scheckel et al., 2012). Overall, the balance of activation and repression between IFE-1 and IFE-3, and how they are recruited to specific germline mRNAs by specific RBPs, is only just beginning to be uncovered. Exploring this pathway in detail will improve our understanding of the diverse modes of post-transcriptional regulation used in different regions of the germline.

IFET-1 is the *C. elegans* homologue of the eIF4E-transporter. IFET-1 contributes to germline development in the distal mitotic end, oocytes maturation, 1-cell embryo development, and P-granule formation (Li et al., 2009; Guven-Ozkan et al., 2010; Sengupta et al., 2013). *ifet-1* loss of function mutant hermaphrodites are sterile unlike mutant males. Sterility is due to defects in meiotic progression. They also showed a germline masculinization phenotype (Sengupta et al., 2013). IFET-1 was also shown to be required for localization of P-granule components such as CGH-1, CAR-1, and PGL-1. Overall, IFET-1 is thought to mediate repression of mRNAs in P-granules in addition to its repressive effects in other regions of the germline.

### Argonautes contribute to post-transcriptional regulation of germline mRNAs

The Argonuate family of RNA-binding proteins is conserved from human to bacteria and archaea (Hutvagner and Simard, 2008). Argonautes are essential for the biogenesis and functionality of several small RNA pathways found in the germline (Weiser and Kim, 2019). These RBPs associate with small RNAs such as microRNAs which are 21–24 nucleotide transcripts that mediate gene expression silencing (Bartel, 2004). In *C. elegans*, primary miRNA transcripts are cleaved by DRSH-1 to make the precursor miRNAs which then get exported to the cytoplasm where they undergo processing by DCR-1 to form the mature miRNA transcript. ALG-1 then associates with the mature miRNA to form the miRISC (miRNA Induced Silencing Complex) to which additional proteins can bind. The miRNA functions as a guide that leads the complex to the target mRNA by binding to its complementary sequence in the UTR of the mRNA. Both ALG-1 and ALG-2 are involved in germline development. Loss of function mutants of either *alg-1* or *alg-2* exhibit reduced fertility. These animals have a small mitotic region and fewer viable oocytes due to increased apoptosis. Both ALG-1 and ALG-2 are expressed in the distal tip cell (DTC) which provides the niche for the mitotically dividing germ cells in the distal end (Bukhari et al., 2012). Animals lacking the miRNAs *let-7*, *lin-4*, *mir-237*, *mir-247*, or *mir-359* exhibit reduced fertility, a shortened mitotic region, and contain few oocytes. Using a pull-down approach followed by small RNA-seq and mass spectrometry, *mir-84* was demonstrated to bind and target the 3′UTR of *gld-1*. Mutating the binding site of *mir-84* in the *gld-1* 3′UTR using CRISPR/Cas9 leads to reduction in the abundance of *mir-84* bound to the 3′UTR (Theil et al., 2019). More recently, a third germline expressed miRNA-associated argonaute termed ALG-5 was identified (Brown et al., 2017). ALG-5 is the fifth member of the *C. elegans* AGO subfamily of argonautes. It was identified by phylogenetic analysis using *C. elegans*, *D. Melanogaster*, and *H. sapiens* argonaute sequences. ALG-5 is expressed in the germline in the cytoplasm as well as the P-granules and its loss leads to reduced fertility. ALG-5 appears to control the timing of oogenesis. Animals deficient in *alg-5* exhibit premature onset of oogenesis (Brown et al., 2017). Although miRNAs that associate with ALG-5 have been identified, the mechanisms of how ALG-5 and its associated miRNAs contribute to germline development and oogenesis remain unknown.

In the embryo, the *mir-35–42* family contributes to proper embryonic and post-embryonic development. Animals that lack this family of miRNAs produce defective embryos with various phenotypes. The embryonic lethality phenotype becomes more severe at elevated temperatures (McJunkin and Ambros, 2014). Moreover, the *mir-35* family is thought to play a role in sex determination in the early embryo by regulating RNA-binding proteins such as SUP-26 and NHL-2 through their 3′UTR (McJunkin and Ambros, 2017). However, the exact mechanism of post-transcriptional regulation is unknown although de-adenylation appears to be involved in the miRNA-mediated post-transcriptional regulation of embryonic mRNAs (Wu et al., 2010). The *mir-51* family functions in late embryos to regulate pharyngeal development (Shaw et al., 2010). Interestingly, *mir-35* and *mir-51* are sufficient to rescue embryonic lethality that occurs in animals lacking Drosha and Pasha (Dexheimer et al., 2020). As such, these two miRNAs are likely the only miRNAs required prior to hatching of L1 larvae. Overall, miRNA-associated argonautes and two specific miRNA families contribute to germline development and embryogenesis. Their most important targets, and how miRNA regulation intersects with RBP-driven regulation, remains to be uncovered.

### P-granules and their role in germline and embryo development

P-granules are membrane-less phase separated organelles that consist of RNAs and proteins (Brangwynne et al., 2009). P-granules are found throughout the germline where they are mainly perinuclear in early meiotic germ cells and then become more cytoplasmic in late oocytes (Updike and Strome, 2010). In the developing embryo, they start diffuse and cytoplasmic throughout the 1-cell embryo and then segregate to the germline precursor cells. PGL and GLH proteins are the core components of P-granules although dozens of other proteins have been shown to also associate with these condensates (Gruidl et al., 1996; Kawasaki et al., 1998; Kuznicki et al., 2000; Kawasaki et al., 2004). These proteins are essential to formation and maintenance of P-granules. While single *pgl-1* or *pgl-3* mutant animals are fertile, they become sterile at elevated temperatures. *pgl-1; pgl-3* double mutant animals are sterile at both normal and elevated temperatures and exhibit defects in the germline (Kawasaki et al., 2004). Knockdown of *pgl-1*, *pgl-3*, *glh-1*, and *glh-4* simultaneously have been shown to cause sterility (Updike et al., 2014). The sterility was due to trans-differentiation of the germ cells to somatic cells. Among the functions of P-granules is post-transcriptional regulation and mRNA surveillance mediated by argonaute proteins. Many of the RNA-binding proteins discussed in this review also localize to P-granules (Schisa et al., 2001). Interestingly, components of the polyadenylation complex including GLD-2 and GLD-4 as well as the translation initiation factor IFE-1 also localize to P-granules suggesting that post-transcriptional regulatory mechanisms occurs in these organelles (Amiri et al., 2001; Wang L. et al., 2002; Schmid et al., 2009; Updike and Strome, 2010). While segregation of P-granules contributes to germline cell fate specification in the early embryo, germ cell fate specification can occur successfully even when P-granules are segregated symmetrically among the soma and germline blastomeres (Gallo et al., 2010). P-granules tend to contain mRNA transcripts with low ribosomal occupancy. It is thought that repressed mRNAs shuttle to P-granules and then get targeted to the germ cell precursor where their translation may be necessary for germ cell fate specification in the embryo (Parker et al., 2020).

## Conclusion and perspectives

By combining our knowledge of the spatiotemporal expression of germline RBPs and their proposed regulatory mechanisms, we find that potentially each region in the germline contains several RBPs that mediate post-transcriptional regulation of hundreds of target mRNAs (Figure 2). Some of these target mRNAs encode RBPs that function in a different region in the germline or even in the same region. Although our knowledge of *C. elegans* germline RNA-binding proteins in terms of their RNA-binding domains, binding specificity, and target mRNAs has expanded tremendously over the past few decades, we still do not know the binding specificity of many germline RNA-binding proteins such as DAZ-1, RNP-8, or GLD-3. We do not know whether the binding sites determined using *in vitro* methods are relevant *in vivo* for germline development, and in most cases the mechanisms of RBP regulation remain incompletely described. Dozens of RBPs are present in the same vicinity in various regions of the germline, but we lack the understanding of which RBPs function together on the same target mRNAs and how one RBP gains priority over another. It is possible that the concentration of each RBP and their intrinsic affinity for their sequence motifs are the primary determinants of target selection. If so, statistical effects tell us that the abundance of functional motifs in any given 3′UTR can strongly influence occupancy. However, the kinetics of RNA association and dissociation may very well contribute, and RNA structure could also play an important role. The biochemical basis of RNA target selection remains undefined for the germline RBPs, and what renders one site functional and another irrelevant, even within the same transcript, remains a mystery.

By combining the well-established *in vitro* methods used to determine binding specificity with modern genome editing methods such as CRISPR/Cas9, it is now possible to determine the relevance of individual RBP binding sites in the 3′UTR of the most critical maternal mRNAs. One challenge is that many of these binding sites are very short, appear in multiple copies in a single 3′UTR, and may overlap with binding sites for other RBPs. Initially, large 3′UTR deletions can be made in the endogenous locus to define the overall importance of any given 3′UTR to germline development. Smaller deletions and/or single base mutations can be made to further dissect the 3′UTR and the contribution of its different regions to the mRNA expression pattern and function in the germline once its importance is established. The 3′UTR mutations can also be made in strains in which the gene of interest is tagged at the endogenous locus with a fluorescent protein to allow for the visual determination of the impact of the 3′UTR mutations on the spatiotemporal expression pattern of the protein. To delineate the network of RBP-mRNA found in each region of the germline, sequencing-based methods such as RIP-seq and CLIP-seq have been instrumental to both define binding motifs and the occupancy of motif-containing mRNAs in worm extracts. These approaches have the advantage of simultaneously mapping the motif as well as detecting interactions that occur in animals or extracts. Additionally, RNA-centric approaches such as interactome capture can be used to identify novel proteins bound to a particular transcript to provide us with information on which RBPs act on a single 3′UTR, and how these interactions change upon mutation. The recently developed method vIPR (*in Vivo* Interaction by Pulldown of RNA), which entails crosslinking proteins and RNA, RNA pull down, and mass spectrometry, can be used to identify proteins as well as small RNAs bound to an mRNA of interest (Theil et al., 2019). Methods such as single molecular RNA-FISH (smRNA-FISH) can provide information about the spatial pattern of the RNA transcripts that compose these RBP-mRNA networks. Together, all these methods can provide tremendous insights into how RBP-mRNA networks function to control germline development and reproduction.

In summary, binding does not always predict regulation. Defining the rules that distinguish functional binding events from non-functional events is critical to describing how germline RBPs orchestrate proper germ cell and embryo development. Since many of the germline RBPs are highly conserved across metazoans, the rules defined in this species may well apply to others. The technology now exists to make targeted deletions and substitutions at the endogenous locus (Kim and Colaiacovo, 2019; Vicencio and Ceron, 2021). Combined with high throughput cataloging of RNA-protein networks and identification of novel RBPs, it will be possible to complete the wiring diagram of maternal mRNA regulation, a tremendous step towards understanding how information flows from parent to progeny in metazoans.
